# Association between anthropometric indices and cardiometabolic risk factors in pre-school children

**DOI:** 10.1186/s12887-015-0500-y

**Published:** 2015-11-06

**Authors:** Juan C. Aristizabal, Jacqueline Barona, Marcela Hoyos, Marcela Ruiz, Catalina Marín

**Affiliations:** School of Nutrition and Dietetics, Universidad de Antioquia UdeA, Calle 70 No. 52-21, Medellín, Colombia; Basic and Applied Microbiology Research Group (MICROBA), School of Microbiology, Program of Ophidism, Universidad de Antioquia UdeA, Calle 70 No. 52-21, Medellín, Colombia; Unit of Food Security, Secretary of Social Inclusion and Family, Alcaldía de Medellín, Colombia

**Keywords:** Childhood, Pre-school children, Obesity, Cardiovascular risk factors, Insulin resistance, Body mass index, Waist circumference, Waist to height ratio, Skinfold thickness, Fat mass index

## Abstract

**Background:**

The world health organization (WHO) and the Identification and prevention of dietary- and lifestyle-induced health effects in children and infants- study (IDEFICS), released anthropometric reference values obtained from normal body weight children. This study examined the relationship between WHO [body mass index (BMI) and triceps- and subscapular-skinfolds], and IDEFICS (waist circumference, waist to height ratio and fat mass index) anthropometric indices with cardiometabolic risk factors in pre-school children ranging from normal body weight to obesity.

**Methods:**

A cross-sectional study with 232 children (aged 4.1 ± 0.05 years) was performed. Anthropometric measurements were collected and BMI, waist circumference, waist to height ratio, triceps- and subscapular-skinfolds sum and fat mass index were calculated. Fasting glucose, fasting insulin, homeostasis model analysis insulin resistance (HOMA-IR), blood lipids and apolipoprotein (Apo) B-100 (Apo B) and Apo A-I were determined. Pearson’s correlation coefficient, multiple regression analysis and the receiver-operating characteristic (ROC) curve analysis were run.

**Results:**

51 % (*n* = 73) of the boys and 52 % (*n* = 47) of the girls were of normal body weight, 49 % (*n* = 69) of the boys and 48 % (*n* = 43) of the girls were overweight or obese. Anthropometric indices correlated (*p* < 0.001) with insulin: [BMI (*r* = 0.514), waist circumference (*r* = 0.524), waist to height ratio (*r* = 0.304), triceps- and subscapular-skinfolds sum (*r* = 0.514) and fat mass index (*r* = 0.500)], and HOMA-IR: [BMI (*r* = 0.509), waist circumference (*r* = 0.521), waist to height ratio (*r* = 0.296), triceps- and subscapular-skinfolds sum (*r* = 0.483) and fat mass index (*r* = 0.492)]. Similar results were obtained after adjusting by age and sex. The areas under the curve (AUC) to identify children with insulin resistance were significant (*p* < 0.001) and similar among anthropometric indices (AUC > 0.68 to AUC < 0.76).

**Conclusions:**

WHO and IDEFICS anthropometric indices correlated similarly with fasting insulin and HOMA-IR. The diagnostic accuracy of the anthropometric indices as a proxy to identify children with insulin resistance was similar. These data do not support the use of waist circumference, waist to height ratio, triceps- and subscapular- skinfolds sum or fat mass index, instead of the BMI as a proxy to identify pre-school children with insulin resistance, the most frequent alteration found in children ranging from normal body weight to obesity.

## Background

The high prevalence of childhood obesity is a public health problem worldwide. The WHO estimated that 42 million children under the age of five were overweight or obese around the world in 2013 [1]. Obesity in school children and adolescents is associated with cardiometabolic risk factors, such as hypertension, hyperlipidemia, insulin resistance and type 2 diabetes [2, 3]. There is less information about the associations of these cardiometabolic risk factors with obesity in pre-schoolers. Likewise, it remains unclear which of the available anthropometric indices has the strongest association with cardiometabolic risk factors at this age [4, 5].

The WHO released growth reference data for children and cut-offs for BMI in 2006 and for triceps- and subscapular-skinfold thickness in 2007 [6, 7]. BMI is the most commonly used anthropometric indicator to assess obesity. However, BMI is based on weight and height and it does not allow differentiating whether an excess of body weight reflects increases of fat mass or fat free mass [2, 8]. Particularly in children, there is a high variability in the fat mass content for a given BMI [9, 10]. By the other hand, skinfold thickness is a more accurate indicator of body fatness than BMI [11, 12]. However, skinfold thickness is more related to subcutaneous fat than to intra-abdominal fat, the last one that more strongly associates with cardiovascular risk [13, 14].

The IDEFICS study in 2014 released reference values and cut-offs for waist circumference, waist to height ratio and fat mass index in children [15]. Waist circumference is an indicator of central adiposity in adults [13, 14] but its accuracy in children may be affected by their growing process [16]. The waist to height ratio appears to be a more sensitive indicator than BMI to identify individual at increased cardiometabolic risk [13, 17]. However, little is known about the utility of this ratio in very young children. The fat mass index is calculated by dividing body fat mass by the square of height in meters. Thus, the fat mass index is expressed in the common units of the BMI and has the advantage of being a measurement of body composition.

Limited studies have compared the associations of anthropometric indices with cardiometabolic risk factors in pre-school children and the results of those studies are conflicting. Garemo et al. in 4 year old Swedish children, failed to find association between BMI and total cholesterol, triglycerides, fasting insulin or the HOMA-IR [5]. Similar results were reported by others [18, 19]. In contrast, Williams et al. found that obesity assessed by BMI was positively associated with triglycerides and negatively with high density lipoprotein cholesterol (HDL-C) in pre-school children living in New York [20]. Similarly, Shea et al. evaluating healthy 2- to 3-year old Hispanic children found that BMI and skinfold thickness positively correlated with fasting insulin but no with C-reactive protein [21].

The actual prevalence of overweight and obesity in pre-school children urges to find an indicator that helps to detect kids with cardiometabolic risk factors at this early age. Recently, WHO (2007) and IDEFICS (2014) released growth reference data and cut-offs for several anthropometrics indices obtained from normal weight children. The current study aimed to examine the relationship of five anthropometric indices with cardiometabolic risk factors in pre-school children, ranging from normal weight to obesity. Additionally, the study compared the sensitivity of these indices to identify children with insulin resistance, the most common cardiometabolic risk factor found in this sample of kids.

## Methods

### Study design

This is a cross-sectional analytical study.

#### Participants

Our study sample was recruited from children attended by the program “Buen Comienzo”, administered by the Secretary of Social Inclusion and Family from the Mayor's office from the city of Medellín-Colombia. Children 2 to 5 year old, free from any physical or psychological condition affecting normal growth were eligible. Children who were sick at the moment of evaluation or were under treatment with steroids or other kind of hormones or medications were excluded. In 2014, three hundred children from the program “Buen Comienzo” were invited to participate in the study; 150 were overweight or obese and 150 were of normal body weight, according to the BMI growth standards and cut-offs by the WHO [6]. Two hundred and fifty two children accepted the invitation, 20 were excluded by not having all the cardiometabolic risk factors measurements; thus, the final sample was 232. This sample size assuming a power of 85 %, at the 95 % level of confidence and z-score of 1.96, allows to detect a minimum correlation between anthropometric indices and HOMA-IR of 0.20 [22], this correlation is lower than others reported by previous studies [4, 18]. The study was performed according to the Helsinki Declaration and was approved by the Bioethical Review Board of the Secretary of Health of Medellin. Informed consent was obtained from all guardians of the children.

#### Anthropometric indices

Anthropometric measurements were performed in duplicate by experienced and trained nutritionists. Body weight, height, triceps- and subscapular skinfold thickness were measured following the technique described by Lohman et al. [23]. Weight was measured to the nearest 0.1 kg using a digital scale (Seca 813, California, USA). Height was measured to the nearest 0.1 cm using a wall mounted mechanical measuring tape (Seca 206, California, USA). Waist circumference was measured to the nearest 0.1 cm, midway between the lowest rib margin and the iliac crest, using a flexible tape (Lufkin W606PM, Maryland, USA). Waist to height ratio was calculated by dividing waist circumference in centimeters by height in centimeters. Triceps- and subscapular-skinfold thickness were measured on the right side of the body, to the nearest millimeter with a caliper (Slim Guide, Miami, USA). Triceps- and subscapular-skinfolds were summed (SF) and the percentage of fat mass (%FM) was calculated with the Slaughter’s equation [24]. In boys; if SF ≤ 35 mm, %FM = 1.21SF – 0.008SF^2^ - 1.7; if SF > 35 mm, %FM = 0.783SF + 1.6. In girls, if SF ≤ 35 mm, %FM = 1.33SF – 0.013SF^2^ - 2.5, if SF > 35 mm, %FM = 0.546SF + 9.7. The fat mass index was obtained by dividing body fat mass by the square of height in meters.

#### Cardiometabolic risk factors

Participants were instructed to fast overnight for 10 to 12 h. Blood was drawn from the antecubital vein in EDTA tubes. Blood was immediately centrifuged at 1500 x g for 15 min at 4 °C. Plasma was aliquoted and kept frozen at −80 °C for further analysis. Plasma glucose and lipids were measured by colorimetric and enzymatic methods using an automatic analyzer (Roche, Cobas c501, Mannheim, Germany). Insulin was measured by chemiluminescence, Apo A-I and Apo B by immunoturbidimetric methods using the same automatic analyzer (Roche, Cobas c501, Mannheim, Germany). HOMA-IR was calculated as plasma glucose (mmol/L) x plasma insulin (mU/l)/22.5 [25]. Individuals with an IDEFICS percentile ≥ 90 for HOMA-IR were classified with insulin resistance [26].

#### Statistical analysis

The data are presented as means ± standard error. Normal distribution of the data was tested with the Kolmogorov-Smirnov test. Anthropometric variables, blood lipids and apolipoproteins (Apo A-I and Apo B) were non-normally distributed and were log-transformed. Unpaired T-student test was used to compare boys and girls. Pearson’s correlation coefficient was used to test associations among anthropometric indices, blood lipids and apolipoproteins. Multiple regression models for log-transformed fasting insulin, HOMA-IR and triglycerides (as dependent variables) were run. The adjusted R^2^ was calculated to determine which of the anthropometric indices has the strongest association with the cardiometabolic risk factors. Receiver-operating characteristic (ROC) curve analysis was run to test the diagnostic accuracy of the anthropometric indices as a proxy to identify children with insulin resistance. p ≤ 0.05 was considered statistically significant.

## Results

A total of 232 children (142 boys and 90 girls) were included in the study (Table 1). Girls were slightly older than boys (4.3 ± 0.07 *vs* 4.1 ± 0.06 y, *p* < 0.05, respectively). BMI-for-age classified 51.4 % (*n* = 73) of the boys and 52.2 % (n = 47) of the girls with normal body weight, 9.2 % (*n* = 13) of the boys and 17.8 % (*n* = 16) of the girls with overweight and 39.4 % (*n* = 56) of the boys and 30.0 % (*n* = 27) of the girls with obesity. The triceps- and subscapular-skinfolds sum was higher (*p* < 0.05) in girls (22.9 ± 1.0 mm) than in boys (20.4 ± 0.7 mm). The glucose levels were higher (*p* < 0.001) in boys (4.68 ± 0.03 mmol/L) than in girls (4.49 ± 0.03 mmol/L). The proportion of children with insulin resistance was similar (*p* = 0.210) in boys 43.0 % (*n* = 61) and girls 38.9 % (*n* = 35).

**Table 1:** Participant characteristics by gender^a^

	All (*n* = 232)		Girls (*n* = 90)		Boys (*n* = 142)		*p*-value^b^
Age (years)	4.1	(0.05)	4.3	(0.07)	4.1	(0.06)	0.032*
Weight (kg)	20.2	(0.31)	20.0	(0.50)	20.0	(0.39)	0.984
Height (cm)	104.6	(0.40)	104.6	(0.63)	104.6	(0.51)	0.886
Height for age z-score^c^	0.18	(0.05)	0.10	(0.08)	0.23	(0.07)	0.244
Body mass index (kg/m^2^)	18.2	(0.22)	18.2	(0.36)	18.2	(0.27)	0.918
Body mass index for age z-score^c^	1.70	(0.13)	1.56	(0.19)	1.77	(0.17)	0.423
Waist circunference (cm)	56.3	(0.50)	56.5	(0.83)	56.0	(0.62)	0.624
Waist to height ratio	0.54	(0.01)	0.54	(0.01)	0.53	(0.01)	0.448
Triceps- and subscapular-skinfolds sum (mm)	21.4	(0.60)	22.9	(1.00)	20.4	(0.73)	0.032*
Fat mass index (kg/m^2^)	3.7	(0.13)	3.9	(0.20)	3.7	(0.16)	0.434
Fasting glucose (mmol-L)	4.60	(0.02)	4.49	(0.03)	4.68	(0.03)	0.000*
Fasting insulin (pmol-L)	44.1	(1.91)	47.7	(3.30)	41.8	(2.31)	0.090
HOMA-IR	1.32	(0.06)	1.4	(0.10)	1.27	(0.07)	0.245
Triglycerides (mmol-L)	1.09	(0.03)	1.03	(0.04)	1.13	(0.04)	0.206
Total cholesterol (mmol-L)	4.33	(0.05)	4.39	(0.09)	4.30	(0.06)	0.414
HDL cholesterol (mmol-L)	1.18	(0.02)	1.21	(0.03)	1.16	(0.03)	0.120
LDL cholesterol (mmol-L)	2.81	(0.05)	2.85	(0.09)	2.78	(0.06)	0.509
Non-HDL cholesterol (mmol-L)	3.15	(0.05)	3.17	(0.09)	3.14	(0.06)	0.978
apoA-I (g-L)	1.26	(0.01)	1.26	(0.02)	1.25	(0.02)	0.503
apoB (g-L)	0.80	(0.01)	0.80	(0.02)	0.79	(0.01)	0.923
apoB/apoA-I	0.66	(0.01)	0.65	(0.02)	0.66	(0.02)	0.707
LDL cholesterol/apoB	3.50	(0.02)	3.54	(0.03)	3.47	(0.03)	0.169

^a^Data presented as mean followed by standard error in parentheses

^b^ *p*-value refer to differences between gender as derived from *T*-test

^c^Calculated from the World Health Organization reference values

*P < 0.05

There were moderate to high correlations between anthropometric indices (Table 2). BMI highly correlated with the fat mass index (*r* = 0.957, *p* < 0.01), the triceps- and subscapular-skinfolds sum (*r* = 0.911, *p* < 0.01) and the waist circumference (*r* = 0.808, *p* < 0.01). BMI moderately correlated with the waist to height ratio (*r* = 0.682, *p* < 0.01). Similar results were observed when correlations where run separately in boys and girls (data not shown).

**Table 2:** Person´s correlation coefficients among anthropometric indices

	Waist circumference	Waist to height ratio	Triceps- and subscapular-skinfolds sum	Fat mass index
Body mass index	0.808**	0.682**	0.911**	0.957**
Waist circunference		0.900**	0.767**	0.791**
Waist to height ratio			0.628**	0.657**
Triceps- and subscapular-skinfolds sum				0.988**

**P < 0.01

Anthropometric indices correlated at different levels with fasting insulin, HOMA-IR and triglycerides (Table 3). The higher correlations were observed among BMI, waist circumference, triceps- and subscapular-skinfolds sum and fat mass index with fasting insulin and HOMA-IR. There were no significant correlations between anthropometric indices and other cardiometabolic risk factors, except for a very low correlation between HDL-C and waist to height ratio (Table 3). Analysis by gender showed similar results, but only in girls the anthropometric indices correlated with triglycerides (Table 3).

**Table 3:** Pearson´s correlation coefficients among anthropometric indices and cardiometabolic risk factors

Overall	Body mass index	Waist circumference	Waist to height ratio	Triceps- and subscapular-skinfolds sum	Fat mass index
Fasting glucose	0.149*	0.177**	0.044	0.090	0.115
Fasting insulin	0.514***	0.524***	0.304***	0.494***	0.500**
HOMA-IR	0.509***	0.521***	0.296***	0.483***	0.492**
Triglycerides	0.230***	0.191**	0.177**	0.168*	0.190**
Total cholesterol	0.001	0.037	−0.072	0.086	0.063
LDL cholesterol	0.019	0.051	−0.037	0.088	0.072
HDL cholesterol	−0.114	−0.092	−0.196**	−0.029	−0.060
Non-HDL cholesterol	0.053	0.082	0.015	0.110	0.097
ApoA-I	−0.024	−0.013	−0.119	0.045	0.022
ApoB	−0.002	0.039	−0.011	0.052	0.040
ApoB/apoA-I	0.012	0.037	0.058	0.015	0.019
LDL cholesterol/apoB	0.013	0.007	−0.058	0.079	0.061
Girls					
Fasting glucose	0.261*	0.281**	0.149	0.204	0.221*
Fasting insulin	0.543***	0.505***	0.332**	0.509***	0.520**
HOMA-IR	0.541***	0.506***	0.330**	0.504***	0.516**
Triglycerides	0.439***	0.367***	0.317**	0.354**	0.382**
Total cholesterol	−0.024	0.008	−0.082	0.049	0.028
LDL cholesterol	−0.011	−0.008	−0.069	0.060	0.040
HDL cholesterol	−0.211*	−0.146	−0.220*	−0.169	−0.187
Non-HDL cholesterol	0.046	0.054	−0.007	0.107	0.092
ApoA-I	−0.095	−0.035	−0.122	−0.052	−0.069
ApoB	−0.012	0.015	−0.022	0.063	0.042
ApoB/apoA-I	0.043	0.031	0.050	0.078	0.070
LDL cholesterol/apoB	−0.059	−0.110	−0.155	−0.014	−0.029
Boys					
Fasting glucose	0.084	0.134	0.013	0.090	0.089
Fasting insulin	0.504***	0.537***	0.285**	0.474***	0.484**
HOMA-IR	0.493***	0.531***	0.275**	0.465***	0.475**
Triglycerides	0.109	0.096	0.119	0.090	0.100
Total cholesterol	0.019	0.054	−0.071	0.097	0.078
LDL cholesterol	0.040	0.087	−0.024	0.097	0.087
HDL cholesterol	−0.055	−0.066	−0.194*	0.026	−0.003
Non-HDL cholesterol	0.058	0.104	0.028	0.109	0.100
ApoA-I	0.023	−0.002	−0.122	0.095	0.070
ApoB	0.004	0.056	−0.005	0.045	0.039
ApoB/apoA-I	−0.010	0.043	0.064	−0.019	−0.010
LDL cholesterol/apoB	0.051	0.063	−0.026	0.104	0.093

*P < 0.05; **P < 0.01; ***P < 0.001

The results of the multiple linear regression for fasting insulin, HOMA-IR and triglycerides are presented in Table 4. After adjusting by age and sex, the BMI explained the higher proportion of the variance in fasting insulin and HOMA-IR in the whole group. The waist circumference, the triceps- and subscapular-skinfolds sum and the fat mass index showed similar results, and just predicted a slight lower proportion of the variance in fasting insulin and HOMA-IR (Table 4). Only in girls, the anthropometric indices explained a low proportion of the variance in triglycerides. The waist to height ratio predicted the lower proportion of the variance of the cardiometabolic factors in both genders.

**Table 4:** Multiple linear regression using as dependent variable the log-transformed values of selected cardiometabolic risk factors

	Insulin		HOMA-IR		Triglycerides	
Overall^a^	B-coefficients ± SE	R^2^	B-coefficients ± SE	R^2^	B-coefficients ± SE	R^2^
Body mass index	1.811 ± 0.189***	0.342	1.877 ± 0.199***	0.333	0.502 ± 0.140**	0.048
Waist circunference	2.308 ± 0.269***	0.303	2.402 ± 0.283***	0.298	0.613 ± 0.196**	0.036
Waist to height ratio	1.527 ± 0.254***	0.203	1.575 ± 0.267***	0.196	0.483 ± 0.174**	0.027
Triceps- and subscapular-skinfolds sum	0.704 ± 0.084***	0.297	0.727 ± 0.088***	0.289	0.174 ± 0.061**	0.029
Fat mass index	0.565 ± 0.064***	0.313	0.584 ± 0.068***	0.305	0.144 ± 0.047**	0.034
Boys^b^						
Body mass index	1.880 ± 0.255***	0.327	1.914 ± 0.267***	0.317	0.254 ± 0.197	0.000
Waist circunference	2.488 ± 0.361***	0.303	2.547 ± 0.377***	0.297	0.326 ± 0.274	0.000
Waist to height ratio	1.425 ± 0.319***	0.181	1.442 ± 0.332***	0.177	0.313 ± 0.223	0.000
Triceps- and subscapular-skinfolds sum	0.709 ± 0.111***	0.277	0.722 ± 0.116***	0.270	0.080 ± 0.133	0.000
Fat mass index	0.562 ± 0.083***	0.295	0.572 ± 0.087***	0.287	0.075 ± 0.063	0.000
Girls^b^						
Body mass index	1.721 ± 0.280***	0.342	1.831 ± 0.298***	0.344	0.855 ± 0.186***	0.179
Waist circunference	2.050 ± 0.402***	0.279	2.200 ± 0.429***	0.283	1.016 ± 0.266***	0.127
Waist to height ratio	1.753 ± 0.429***	0.213	1.870 ± 0.458**	0.216	0.857 ± 0.279**	0.080
Triceps- and subscapular-skinfolds sum	0.698 ± 0.129***	0.305	0.736 ± 0.138***	0.304	0.314 ± 0.088**	0.111
Fat mass index	0.573 ± 0.102***	0.318	0.607 ± 0.109***	0.318	0.271 ± 0.069***	0.132

*p < 0.05; **p < 0.01; ***p < 0.001 refers to the level of significance of the change in the cardiometabolic risk factor per unit of change in the anthropometric index

^a^Adjusted by age and sex. ^b^Adjusted by age

**Fig. 1:**
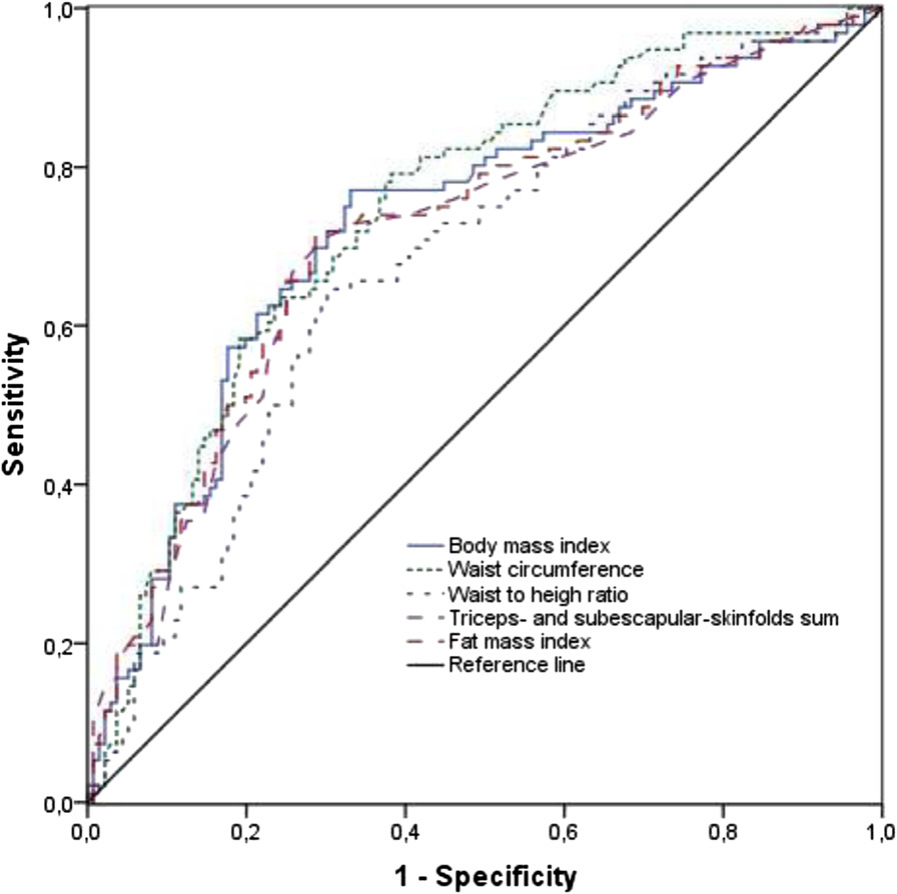
Sensitivity of the anthropometric indices to identify children with insulin resistance. Receiver operating characteristic curves for anthropometric indices in the detection of insulin resistance, using a homeostatic model assessment insulin resistance. (*n* = 96)

ROC analysis showed that anthropometric indices performed fairly detecting children with insulin resistance (Fig. 1). The area under the curve (AUC) was only marginally better for waist circumference [AUC = 0.75 (95 % CI: 0.68-0.81), *p* = 0.000] than for BMI [AUC = 0.73 (95 % CI: 0.66-0.80), *p* = 0.000], fat mass index [AUC = 0.72 (95 % CI: 0.65-0.79), *p* = 0.000], triceps- and subscapular-skinfolds sum [AUC = 0.71 (95 % CI: 0.64-0.78), *p* = 0.000] and, waist-to-high ratio [AUC = 0.68 (95 % CI: 0.61-0.75) *p* = 0.000].

## Discussion

The purpose of the study was to examine the relationship between several anthropometric indices and cardiometabolic risk factors in pre-school children ranging from normal weight to obesity. Anthropometric indices endorsed by WHO (BMI, triceps and subscapular skinfolds) and IDEFICS (waist circumference, waist to height ratio, fat mass index) were compared. The main finding was that all anthropometric indices significantly correlated with fasting insulin and HOMA-IR. Additionally, the diagnostic accuracy of the anthropometric indices as a proxy to identify children with insulin resistance was fair and alike. There were very weak or inexistent associations between the anthropometric indices with fasting glucose, blood lipids and apolipoproteins.

Similar to previous studies, the results showed significant associations between anthropometric indices and cardiometabolic risk factors in pre-school children [4, 27]. These findings support the concept that cardiovascular disease start to develop early in life and suggest that anthropometric indices may play a role identifying pre-school children at risk [21, 28, 29]. However, not all studies report these associations [5, 18, 19]. These conflicting results are probably due, at least in part, to differences in the nutritional status of the children evaluated. This study used a sample of children with higher proportion of overweight and obesity (48 %) which probably strength the association between anthropometric indices and cardiometabolic risk factors. This concept is supported by longitudinal studies reporting that increases in BMI percentiles in overweight or obese children reflect mainly gains in fat mass [8, 30]. By the contrary, increases in BMI percentiles in underweight or normal weight children reflect mostly augments in lean body mass [8, 30].

The obesity-related cardiometabolic risk factors (i.e. abnormal lipid profile and insulin resistance) are associated to an excess of central adiposity in adults, but this association is not clearly established in pre-school children [18, 21]. The study found that indices of visceral adiposity (waist circumference and waist-to-height ratio), total fat content (triceps- and subscapular-skinfolds sum and fat mass index) and body weight (BMI) related similarly to fasting insulin and HOMA-IR. Furthermore, there were no significant differences between anthropometric indices to identify children with insulin resistance (Fig. 1). These data suggest that at the early age of pre-school children, the excess of body weight, total fat and abdominal fat, might alter similarly the insulin metabolism.

Anthropometric indices correlated with insulin and insulin resistance, but their associations with other cardiometabolic risk factors was almost inexistent. Additionally, using the reference values from IDEFICS [26, 31] to calculate the frequency of the cardiometabolic risk factors, high insulin levels (40.5 %) and insulin resistance (41.4 %) were the most frequent. These findings support the concept that insulin resistance is an early metabolic alteration, and it probably precedes the appearance of other cardiometabolic risk factors [32–34]. In addition, the results suggest that measurement of insulin resistance should be prioritized in populations of pre-school children with high prevalence of overweight/obesity.

To this point, the study has no clear explanations for the association between anthropometric indices and triglycerides only in girls. A possible reason might be their higher fat depots compared to boys. Girls showed lower blood glucose levels and higher adiposity levels than boys, although the BMI between boys and girls were similar. Thus, at fasting conditions, girls seem to use a higher proportion of blood glucose for basal metabolism preserving fat storages. This may be the result of a higher insulin sensitivity in girls compared to boys as reported in previous studies [35, 36].

The study had some strengths and limitations. Among the strengths: a) the comparison of anthropometric indices endorsed by WHO and IDEFICS, b) the age of the children analyzed, there are few studies in children less than 5 year old [4], and c) the inclusion of children ranging from normal weight to obesity, thus avoiding that the relations between anthropometric indices and cardiometabolic risk factors be altered by underweight children. The limitations were: a) this is a cross-sectional study and does not allow to stablish cause-effect relationships, b) the study model does not provide information about the anthropometric indices ability for predicting future health outcomes, and c) the study sample is not representative of any particular group of the population. Participants were selected with the objective of analyzing how reference values and cut-offs derived from normal weight children are related to cardiometabolic risk factors in a group of children with a high proportion of overweight/obesity.

## Conclusions

The anthropometric indices endorsed by WHO and IDEFICS correlated similarly with fasting insulin and HOMA-IR in pre-school children. BMI provided similar information about cardiometabolic risk factors than waist circumference, waist to height ratio, triceps- and subscapular-skinfolds sum and fat mass index. The BMI has some advantages compared to the other anthropometric indices analyzed, BMI uses body weight and height, simpler measurements than waist circumference and skinfold thickness; BMI has been widely used around the world allowing comparisons between areas and populations over time. The study results do not support the use of waist circumference, waist to height ratio, triceps- and subscapular- skinfolds sum or fat mass index, instead of the BMI as a proxy to identify pre-school children with insulin resistance, the most frequent alteration found in children ranging from normal body weight to obesity.

## Abbreviations

WHO: World health organization
IDEFICS: Identification and prevention of dietary- and lifestyle-induced health effects in children and infants- study
HOMA-IR: Homeostasis model analysis insulin resistance
HDL-C: High density lipoprotein cholesterol
BMI: Body mass index
ROC: Receiver-operating characteristic
AUC: Area under the curve
